# Bacterial meningitis-induced demyelination: A logical fallacy or groundbreaking avenue in neuroscience?

**DOI:** 10.3934/Neuroscience.2023013

**Published:** 2023-06-25

**Authors:** Tjokorda Istri Pramitasuri, Ni Made Susilawathi, AA Raka Sudewi

**Affiliations:** 1 Doctoral Program in Medical Sciences, Faculty of Medicine, Universitas Udayana, Bali, Indonesia 80232; 2 Department of Neurology, Faculty of Medicine, Universitas Udayana, Bali, Indonesia 80232

To the Editor,

Over time, there has been increasing evidence of surviving meningitis patients, especially young adults, having high chances of developing permanent nervous system disability [1]. In this regard, demyelination is hypothesized as one of the emerging pathological mechanisms, since multiple sclerosis (MS), a chronic progressive demyelinating disorder of the central nervous system (CNS), is the most common cause of neurological disability in young adults [2],[3]. Linking demyelination with the etiology of meningitis, neuroscientists have long made efforts to discover the pathogenesis of virus-induced demyelination, while research on the mechanism of bacteria-induced demyelination is scarce.

On the other hand, a recent study from Ma et al. (2023) asserts that the intraperitoneal injection of the toxin epsilon toxin (ETX), which is made by the gut microbiota member *Clostridium perfringens*, causes demyelination in the corpus callosum, thalamus, cerebellum, brainstem and spinal cord in mice, with an experimental autoimmune encephalomyelitis (EAE) model [4]. This finding has been supported by another study which described the detailed pathological mechanisms of ETX, including blood-brain barrier (BBB) disruption [5], induction of inflammatory cytokines in brain parenchyma that exacerbates neuroinflammation and, finally, damaging the oligodendrocytes via elevation of extracellular glutamate along with activation of metabotropic type 1 glutamate receptors (mGluR1) and N-methyl-D-aspartate receptor (NMDAR), which results in demyelination [6],[7]. The toxin, which belongs to the group of cholesterol-dependent cytolysins (CDCs), is known to share the same properties as other CDCs which belong to meningitis-causing bacteria: damaging cell membranes by pore formation and exhibiting cytotoxic effects on various cells [8]. Thus, these findings raise a question: Does bacterial meningitis also potentially cause demyelination?

We discovered evidence in a larger body of literature to demonstrate that other CDCs from meningitis-causing bacteria have similar mechanisms to ETX which, when employed by ETX, induce demyelination in the CNS, although there is still no direct proof that bacterial meningitis can cause demyelination either *in vitro* or *in vivo*. The brain's glutamate level is altered by pneumolysin (PLY) from *Streptococcus pneumoniae*, the most common etiology of bacterial meningitis, which results in synaptic injury [9]. Additionally, an *in vivo* study in pigs has demonstrated that myelin degradation occurred after *Streptococcus suis* was administered. Suilysin (SLY), the toxin produced by this organism, exhibited high expression *in vivo* and was suspected to be the main culprit of the observed pathological event [10]. On the other hand, meningitis-causing bacteria can also activate microglia, while ETX has been reported to only act on oligodendrocytes [11]. However, we should keep an eye on their similarities since the possible pathological mechanisms could be related.

How could this emerging concept impact the hospital clinical setting and academic research? Antibiotics should be used appropriately, and treatments should start at the right time in a clinical setting to stop demyelination from progressing. If the pathogenesis has already been established, a strategic research direction on drug-prospecting studies will be followed by the development of specifically targeted treatment modalities based on the investigated pathogenic processes. We are optimistic that a sound understanding of pathogenesis will be a fruitful avenue to prevent further disabilities for bacterial meningitis patients, which have a significant impact on the patients' quality of life.
